# 5-Hy­droxy-3-phenyl-5-trifluoro­meth­yl-4,5-dihydro-1*H*-pyrazole

**DOI:** 10.1107/S160053681103368X

**Published:** 2011-08-27

**Authors:** Abdullah M. Asiri, Abdulrahman O. Al-Youbi, Hassan M. Faidallah, Seik Weng Ng

**Affiliations:** a Chemistry Department, Faculty of Science, King Abdulaziz University, PO Box 80203 Jeddah, Saudi Arabia; b Center of Excellence for Advanced Materials Research, King Abdulaziz University, PO Box 80203 Jeddah, Saudi Arabia; c Department of Chemistry, University of Malaya, 50603 Kuala Lumpur, Malaysia

## Abstract

The five-membered dihydro­pyrazole ring in the title compound, C_10_H_9_F_3_N_2_O, is approximately planar (r.m.s. deviation 0.111 Å for all non-H atoms) and its phenyl substituent is aligned at an angle of 14.7 (2)°. Adjacent mol­ecules are linked by N—H⋯O and O—H⋯N hydrogen bonds, generating ribbons running along the *b* axis of the monoclinic unit cell.

## Related literature

For the synthesis, see: Yakimovich *et al.* (2002 ▶); Zelenin *et al.* (1995 ▶). For two related structures, see: Dias & Goh (2004 ▶); Yang & Raptis (2003 ▶).
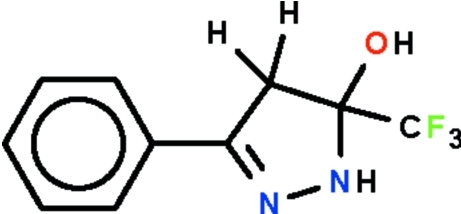


## Experimental

### 

#### Crystal data

C_10_H_9_F_3_N_2_O
*M* *_r_* = 230.19
Monoclinic, 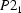

*a* = 9.1000 (6) Å
*b* = 5.4032 (3) Å
*c* = 10.4515 (7) Å
β = 108.139 (7)°
*V* = 488.35 (5) Å^3^
*Z* = 2
Mo *K*α radiation
μ = 0.14 mm^−1^
*T* = 100 K
0.20 × 0.15 × 0.10 mm

#### Data collection

Agilent SuperNova Dual diffractometer with Atlas detector
Absorption correction: multi-scan (*CrysAlis PRO*; Agilent, 2010 ▶) *T*_min_ = 0.972, *T*_max_ = 0.986
4222 measured reflections
1230 independent reflections
1060 reflections with *I* > 2σ(*I*)
*R*_int_ = 0.039

#### Refinement

*R*[*F* ^2^ > 2σ(*F* ^2^)] = 0.037
*wR*(*F* ^2^) = 0.087
*S* = 1.05
1230 reflections
153 parameters
3 restraints
H atoms treated by a mixture of independent and constrained refinement
Δρ_max_ = 0.29 e Å^−3^
Δρ_min_ = −0.26 e Å^−3^

### 

Data collection: *CrysAlis PRO* (Agilent, 2010 ▶); cell refinement: *CrysAlis PRO*; data reduction: *CrysAlis PRO*; program(s) used to solve structure: *SHELXS97* (Sheldrick, 2008 ▶); program(s) used to refine structure: *SHELXL97* (Sheldrick, 2008 ▶); molecular graphics: *X-SEED* (Barbour, 2001 ▶); software used to prepare material for publication: *publCIF* (Westrip, 2010 ▶).

## Supplementary Material

Crystal structure: contains datablock(s) global, I. DOI: 10.1107/S160053681103368X/bt5617sup1.cif

Structure factors: contains datablock(s) I. DOI: 10.1107/S160053681103368X/bt5617Isup2.hkl

Supplementary material file. DOI: 10.1107/S160053681103368X/bt5617Isup3.cml

Additional supplementary materials:  crystallographic information; 3D view; checkCIF report

## Figures and Tables

**Table 1:** Hydrogen-bond geometry (Å, °)

*D*—H⋯*A*	*D*—H	H⋯*A*	*D*⋯*A*	*D*—H⋯*A*
O1—H1⋯N1^i^	0.84 (1)	2.03 (2)	2.833 (3)	162 (4)
N2—H2⋯O1^ii^	0.88 (1)	2.13 (2)	2.974 (3)	161 (3)

Symmetry codes: (i) 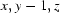; (ii) 
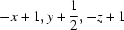
.

**Fig. 1.**
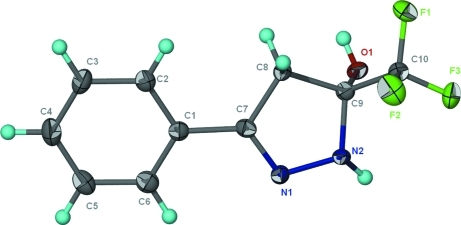
Anisotropic displacement ellipsoid plot (Barbour, 2001) of C10H9F3N2O at the 70% probability level; H atoms are drawn as spheres of arbitrary radius.

**Fig. 2.**
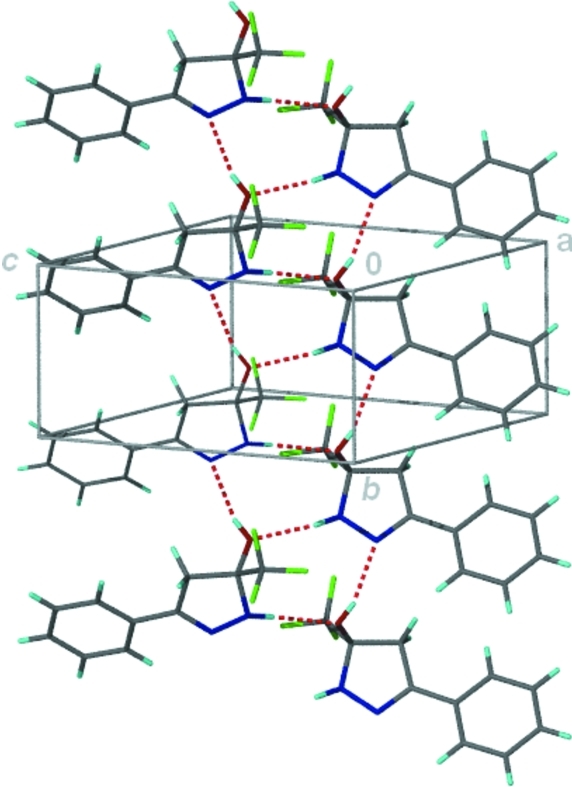
Hydrogen-bonded ribbon structure.

## supplementary crystallographic information

### Comment

We had intended to synthesize 5-phenyl-3-(trifluoromethyl)pyrazole, whose crystal structure has been reported (Dias & Goh, 2004). However, the strongly electron-withdrawing nature of the α,β-diketone used in the synthesis led to the isolation of a stable intermediate, a dihydropyrazole (Scheme I), that when dehydrated, should furnish the pyrazole. The synthesis of the dihydropyrazole has previously been reported (Yakimovich *et al.*, 2002; Zelenin *et al.*, 1995). The five-membered dihydropyrazole ring of C_10_H_9_F_3_N_2_O is approximately planar, the ring being buckled at the methylene carbon, and its phenyl substituent is aligned at 14.7 (2)° (Fig. 1). Adjacent molecules are linked by N—H···O and O—H···N hydrogen bonds (Table 1) to generate a helical chain running along the *b* axis of the monoclinic unit cell (Fig. 2).

The crystal structure of the 2-naphthyl substituted analog has been reported (Yang & Raptis, 2003); both compounds should similar hydrogen-bonding features.

### Experimental

4,4,4-Trifluoro-1-phenyl-1,3-butanedione (10 mmol) in ethanol (50 ml) was refluxed with hydrazine hydrate (10 mmol) for 4 h. Water was added to precipitate the product, which was collected and recrystallized from ethanol; m.p. 415–416 K.

### Refinement

Carbon-bound H-atoms were placed in calculated positions [C—H 0.95 Å, *U*_iso_(H) 1.2*U*_eq_(C)] and were included in the refinement in the riding model approximation.

The amino and hydroxy H-atoms were located in a difference Fourier map, and were refined isotropically with distance restraints of N—H 0.88 (1) Å and O—H 0.84 (1) Å.

In the absence of anomalous scatterers, 727 Friedel pairs were merged.

### Crystal data

**Table:**

C_10_H_9_F_3_N_2_O	*F*(000) = 236
*M**_r_* = 230.19	*D*_x_ = 1.565 Mg m^−^^3^
Monoclinic, *P*2_1_	Mo *K*α radiation, λ = 0.71073 Å
Hall symbol: P 2yb	Cell parameters from 1825 reflections
*a* = 9.1000 (6) Å	θ = 2.4–29.2°
*b* = 5.4032 (3) Å	µ = 0.14 mm^−^^1^
*c* = 10.4515 (7) Å	*T* = 100 K
β = 108.139 (7)°	Prism, colourless
*V* = 488.35 (5) Å^3^	0.20 × 0.15 × 0.10 mm
*Z* = 2	

### Data collection

**Table:**

Agilent SuperNova Dual diffractometer with Atlas detector	1230 independent reflections
Radiation source: SuperNova (Mo) X-ray Source	1060 reflections with *I* > 2σ(*I*)
Mirror	*R*_int_ = 0.039
Detector resolution: 10.4041 pixels mm^-1^	θ_max_ = 27.5°, θ_min_ = 2.4°
ω scans	*h* = −11→11
Absorption correction: multi-scan (*CrysAlis PRO*; Agilent, 2010)	*k* = −6→6
*T*_min_ = 0.972, *T*_max_ = 0.986	*l* = −13→13
4222 measured reflections	

### Refinement

**Table:**

Refinement on *F*^2^	Primary atom site location: structure-invariant direct methods
Least-squares matrix: full	Secondary atom site location: difference Fourier map
*R*[*F*^2^ > 2σ(*F*^2^)] = 0.037	Hydrogen site location: inferred from neighbouring sites
*wR*(*F*^2^) = 0.087	H atoms treated by a mixture of independent and constrained refinement
*S* = 1.05	*w* = 1/[σ^2^(*F*_o_^2^) + (0.0412*P*)^2^ + 0.1359*P*] where *P* = (*F*_o_^2^ + 2*F*_c_^2^)/3
1230 reflections	(Δ/σ)_max_ = 0.001
153 parameters	Δρ_max_ = 0.29 e Å^−^^3^
3 restraints	Δρ_min_ = −0.26 e Å^−^^3^

### Fractional atomic coordinates and isotropic or equivalent isotropic displacement parameters (Å^2^)

**Table:**

	*x*	*y*	*z*	*U*_iso_*/*U*_eq_	
F1	0.15155 (18)	0.5006 (3)	0.41240 (17)	0.0224 (4)	
F2	0.07047 (18)	0.8747 (4)	0.36876 (17)	0.0275 (4)	
F3	0.27744 (19)	0.7556 (3)	0.32684 (15)	0.0239 (4)	
O1	0.4291 (2)	0.6574 (4)	0.59470 (19)	0.0147 (4)	
H1	0.407 (4)	0.534 (5)	0.634 (4)	0.045 (12)*	
N1	0.3514 (3)	1.1846 (4)	0.6715 (2)	0.0148 (5)	
N2	0.3409 (3)	1.0670 (4)	0.5493 (2)	0.0147 (5)	
H2	0.425 (2)	1.094 (6)	0.526 (3)	0.026 (9)*	
C1	0.2622 (3)	1.1255 (5)	0.8657 (3)	0.0147 (6)	
C2	0.1637 (3)	0.9892 (6)	0.9190 (3)	0.0177 (6)	
H2A	0.1048	0.8565	0.8688	0.021*	
C3	0.1519 (3)	1.0472 (6)	1.0446 (3)	0.0209 (6)	
H3	0.0860	0.9528	1.0807	0.025*	
C4	0.2358 (3)	1.2421 (6)	1.1175 (3)	0.0226 (7)	
H4	0.2278	1.2809	1.2037	0.027*	
C5	0.3317 (3)	1.3813 (6)	1.0650 (3)	0.0212 (6)	
H5	0.3879	1.5167	1.1148	0.025*	
C6	0.3455 (3)	1.3230 (5)	0.9398 (3)	0.0188 (6)	
H6	0.4119	1.4179	0.9045	0.023*	
C7	0.2782 (3)	1.0531 (5)	0.7351 (3)	0.0144 (6)	
C8	0.2110 (3)	0.8206 (5)	0.6601 (3)	0.0141 (6)	
H8A	0.0979	0.8347	0.6169	0.017*	
H8B	0.2338	0.6738	0.7197	0.017*	
C9	0.2974 (3)	0.8121 (5)	0.5561 (3)	0.0140 (6)	
C10	0.1981 (3)	0.7364 (5)	0.4152 (3)	0.0167 (6)	

### Atomic displacement parameters (Å^2^)

**Table:**

	*U*^11^	*U*^22^	*U*^33^	*U*^12^	*U*^13^	*U*^23^
F1	0.0249 (9)	0.0185 (9)	0.0224 (8)	−0.0067 (7)	0.0054 (7)	−0.0054 (7)
F2	0.0243 (9)	0.0285 (10)	0.0233 (9)	0.0091 (8)	−0.0019 (7)	0.0000 (8)
F3	0.0318 (9)	0.0273 (10)	0.0154 (8)	−0.0024 (8)	0.0116 (7)	−0.0015 (7)
O1	0.0157 (9)	0.0121 (10)	0.0179 (10)	0.0015 (8)	0.0073 (8)	0.0027 (8)
N1	0.0202 (12)	0.0116 (11)	0.0136 (11)	0.0025 (9)	0.0068 (9)	0.0018 (9)
N2	0.0204 (12)	0.0094 (11)	0.0181 (11)	−0.0013 (10)	0.0116 (9)	−0.0008 (9)
C1	0.0162 (12)	0.0127 (14)	0.0148 (13)	0.0027 (11)	0.0045 (10)	0.0019 (11)
C2	0.0196 (13)	0.0168 (14)	0.0181 (13)	0.0000 (12)	0.0079 (11)	−0.0017 (11)
C3	0.0241 (14)	0.0242 (17)	0.0173 (14)	0.0020 (13)	0.0107 (12)	0.0030 (13)
C4	0.0278 (15)	0.0247 (17)	0.0151 (13)	0.0054 (14)	0.0065 (11)	−0.0013 (13)
C5	0.0220 (14)	0.0196 (15)	0.0212 (15)	0.0026 (13)	0.0054 (12)	−0.0042 (12)
C6	0.0190 (13)	0.0170 (15)	0.0202 (14)	0.0016 (12)	0.0059 (11)	0.0000 (12)
C7	0.0138 (12)	0.0120 (13)	0.0173 (13)	0.0011 (11)	0.0048 (10)	−0.0003 (11)
C8	0.0163 (12)	0.0129 (15)	0.0156 (13)	−0.0001 (11)	0.0086 (10)	0.0000 (11)
C9	0.0145 (12)	0.0130 (15)	0.0155 (13)	0.0013 (11)	0.0061 (10)	0.0002 (11)
C10	0.0209 (14)	0.0141 (15)	0.0154 (13)	0.0002 (12)	0.0061 (10)	0.0007 (12)

### Geometric parameters (Å, °)

**Table:**

F1—C10	1.340 (3)	C2—H2A	0.9500
F2—C10	1.338 (3)	C3—C4	1.382 (4)
F3—C10	1.342 (3)	C3—H3	0.9500
O1—C9	1.413 (3)	C4—C5	1.387 (4)
O1—H1	0.836 (10)	C4—H4	0.9500
N1—C7	1.290 (3)	C5—C6	1.389 (4)
N1—N2	1.403 (3)	C5—H5	0.9500
N2—C9	1.441 (4)	C6—H6	0.9500
N2—H2	0.882 (10)	C7—C8	1.505 (4)
C1—C6	1.396 (4)	C8—C9	1.528 (4)
C1—C2	1.403 (4)	C8—H8A	0.9900
C1—C7	1.471 (4)	C8—H8B	0.9900
C2—C3	1.386 (4)	C9—C10	1.524 (4)
			
C9—O1—H1	107 (3)	C1—C6—H6	119.9
C7—N1—N2	108.6 (2)	N1—C7—C1	123.2 (3)
N1—N2—C9	109.3 (2)	N1—C7—C8	112.5 (2)
N1—N2—H2	111 (2)	C1—C7—C8	124.3 (2)
C9—N2—H2	116 (2)	C7—C8—C9	100.4 (2)
C6—C1—C2	119.0 (3)	C7—C8—H8A	111.7
C6—C1—C7	121.7 (3)	C9—C8—H8A	111.7
C2—C1—C7	119.3 (3)	C7—C8—H8B	111.7
C3—C2—C1	120.4 (3)	C9—C8—H8B	111.7
C3—C2—H2A	119.8	H8A—C8—H8B	109.5
C1—C2—H2A	119.8	O1—C9—N2	111.0 (2)
C4—C3—C2	120.1 (3)	O1—C9—C10	108.2 (2)
C4—C3—H3	120.0	N2—C9—C10	107.4 (2)
C2—C3—H3	120.0	O1—C9—C8	113.2 (2)
C3—C4—C5	120.2 (3)	N2—C9—C8	102.5 (2)
C3—C4—H4	119.9	C10—C9—C8	114.4 (2)
C5—C4—H4	119.9	F2—C10—F1	106.9 (2)
C4—C5—C6	120.1 (3)	F2—C10—F3	107.4 (2)
C4—C5—H5	119.9	F1—C10—F3	107.0 (2)
C6—C5—H5	119.9	F2—C10—C9	112.8 (2)
C5—C6—C1	120.2 (3)	F1—C10—C9	111.4 (2)
C5—C6—H6	119.9	F3—C10—C9	111.1 (2)
			
C7—N1—N2—C9	−18.0 (3)	C1—C7—C8—C9	−166.2 (2)
C6—C1—C2—C3	−1.3 (4)	N1—N2—C9—O1	−95.1 (2)
C7—C1—C2—C3	177.2 (2)	N1—N2—C9—C10	146.8 (2)
C1—C2—C3—C4	0.8 (4)	N1—N2—C9—C8	25.9 (3)
C2—C3—C4—C5	0.3 (4)	C7—C8—C9—O1	96.5 (2)
C3—C4—C5—C6	−1.0 (4)	C7—C8—C9—N2	−23.0 (2)
C4—C5—C6—C1	0.5 (4)	C7—C8—C9—C10	−138.9 (2)
C2—C1—C6—C5	0.6 (4)	O1—C9—C10—F2	−179.4 (2)
C7—C1—C6—C5	−177.8 (2)	N2—C9—C10—F2	−59.5 (3)
N2—N1—C7—C1	−178.3 (2)	C8—C9—C10—F2	53.5 (3)
N2—N1—C7—C8	1.3 (3)	O1—C9—C10—F1	60.5 (3)
C6—C1—C7—N1	−10.1 (4)	N2—C9—C10—F1	−179.7 (2)
C2—C1—C7—N1	171.5 (2)	C8—C9—C10—F1	−66.7 (3)
C6—C1—C7—C8	170.4 (2)	O1—C9—C10—F3	−58.7 (3)
C2—C1—C7—C8	−8.0 (4)	N2—C9—C10—F3	61.1 (3)
N1—C7—C8—C9	14.3 (3)	C8—C9—C10—F3	174.1 (2)

### Hydrogen-bond geometry (Å, °)

**Table:**

*D*—H···*A*	*D*—H	H···*A*	*D*···*A*	*D*—H···*A*
O1—H1···N1^i^	0.84 (1)	2.03 (2)	2.833 (3)	162 (4)
N2—H2···O1^ii^	0.88 (1)	2.13 (2)	2.974 (3)	161 (3)

Symmetry codes: (i) *x*, *y*−1, *z*; (ii) −*x*+1, *y*+1/2, −*z*+1.
